# In Early Breast Cancer, the Ratios of Neutrophils, Platelets and Monocytes to Lymphocytes Significantly Correlate with the Presence of Subsets of Circulating Tumor Cells but Not with Disseminated Tumor Cells

**DOI:** 10.3390/cancers14143299

**Published:** 2022-07-06

**Authors:** Sabine Kasimir-Bauer, Ebru Karaaslan, Olaf Hars, Oliver Hoffmann, Rainer Kimmig

**Affiliations:** 1 Department of Gynecology and Obstetrics, University Hospital Essen, 45147 Essen, Germany; ebru.karaaslan@uk-essen.de (E.K.); oliver.hoffmann@uk-essen.de (O.H.); rainer.kimmig@uk-essen.de (R.K.); 2 Science Consultant, 10781 Berlin, Germany; oh@olafhars-wissenschaft.de

**Keywords:** circulating tumor cells, disseminated tumor cells, neutrophil to lymphocyte ratio, platelet to lymphocyte ratio, monocyte to lymphocyte ratio

## Abstract

**Simple Summary:**

Circulating tumor cells (CTCs) are potential precursors of metastasis and while travelling through the peripheral blood, they crosstalk with different blood cells before a few of them manage to settle down as disseminated tumor cells (DTCs). Little is known about the correlation of blood cells with CTCs/DTCs in early breast cancer (BC). We retrospectively recorded clinical data, results for CTCs, DTCs and blood cell counts from 171 early staged diagnosed BC patients and demonstrated that the presence of epithelial CTCs was related to reduced lymphocyte and monocyte counts, to elevated neutrophil to lymphocyte and platelet to lymphocyte ratios while CTCs in epithelial mesenchymal transition associated with a reduced monocyte to lymphocyte ratio. No significant correlations were found for DTCs, however, DTC-positive patients, harboring a lower platelet to lymphocyte ratio, had a significant shorter overall survival. We confirm that pro-inflammatory markers in blood are closely related to the presence of CTC subtypes, the precursors of metastasis.

**Abstract:**

Circulating tumor cells (CTCs) crosstalk with different blood cells before a few of them settle down as disseminated tumor cells (DTCs). We evaluated the correlation between CTC subtypes, DTCs and the neutrophil to lymphocyte ratio (NLR), platelet to lymphocyte ratio (PLR) and monocyte to lymphocyte ratio (MLR) for better prognostication of 171 early staged diagnosed breast cancer (BC) patients. —Clinical data and blood values before treatment were retrospectively recorded, representing the 75% percentile, resulting in 3.13 for NLR, 222.3 for PLR and 0.39 for MLR, respectively. DTCs were analyzed by immunocytochemistry using the pan-cytokeratin antibodyA45-B/B3. CTCs were determined applying the AdnaTests *BreastCancerDetect* and *EMT (Epithelial Mesenchymal Transition) Detect*. —Reduced lymphocyte (*p* = 0.007) and monocyte counts (*p* = 0.012), an elevated NLR (*p* = 0.003) and PLR (*p* = 0.001) significantly correlated with the presence of epithelial CTCs while a reduced MLR was related to EMT-CTCs (*p* = 0.045). PLR (*p* = 0.029) and MLR (*p* = 0.041) significantly related to lymph node involvement and monocyte counts significantly correlated with OS (*p* = 0.034). No correlations were found for NLR, PLR and MLR with DTCs, however, DTC-positive patients, harboring a lower PLR, had a significant shorter OS (*p* = 0.043). —Pro-inflammatory markers are closely related to different CTC subsets. This knowledge might improve risk prognostication of these patients.

## 1. Introduction

Breast cancer (BC) is the leading cause of cancer associated mortality in women worldwide. Over the years, the prognosis of these patients has steadily improved and current treatment options depend on a variety of factors including age, node status, tumor stage, histological subtype, histological grade and lymphatic-vascular invasion [1].

Although surgical and treatment options have markedly been improved, 20% of early staged diagnosed BC patients show a relapse of the disease, even after 10–20 years after first diagnosis [2,3,4]. One explanation for this phenomenon is early micro-metastatic spread of tumor cells, reflected by circulating tumor cells (CTCs) in blood, the so-called precursors of metastasis. Their prognostic relevance, mostly based on CTC enumeration, has been demonstrated in several clinical studies including thousands of patients [5,6]. However, the metastatic process is complex and not completely understood. While leaving the tumor and travelling through the peripheral blood, some CTCs can change from an epithelial to mesenchymal character, known as epithelial to mesenchymal transition (EMT) which has been associated with an aggressive phenotype [7]. Furthermore, travelling from primary site to secondary organs, CTCs crosstalk with different types of blood cells influencing their behavior and ability to settle down as disseminated tumor cells (DTCs), preferentially the bone marrow (BM) which mostly has been sampled and where the majority of metastatic lesions in BC are located [3].

Having arrived in secondary organs, DTCs have to adapt to new environmental conditions. Although little is known about survival conditions of DTCs in the BM, some DTCs were shown to have stem cell character with the ability of self-renewal [8,9] and we recently demonstrated that early staged diagnosed BC patients harboring DTCs expressing the chemokine receptor type 4 (CXCR4) and the transcription factor JUNB had a higher risk for relapse [10]. Interestingly, a recent publication identified a sub-population of osteoblasts, manipulated in their function by DTCs, the so-called educated osteoblasts, which in turn crosstalk with DTCs via proteins and soluble factors leading to a reduction in BC cell proliferation and metastatic latency [11].

Pro-inflammatory blood cells that might play a role in tumor progression and metastasis include white blood cells (WBC), namely lymphocytes, neutrophils, monocytes as well as platelets. Even more relevant, the neutrophil to lymphocyte ratio (NLR), the platelet to lymphocyte ratio (PLR) as well as the monocyte to lymphocyte ratio (MLR) have been associated with a reduced progression free (PFS) and overall survival (OS) in localized disease, in the neo-adjuvant setting before and/or after chemotherapy as well as in metastatic BC [12]. In recent years, especially neutrophils gained a lot of attention in this context. Although described as short-lived effector cells, they seem to acquire immunosuppressive and pro-tumorigenic functions and their role in cancer has been related to metastasis [13,14]. In this regard, neutrophils were shown to promote cancer cell entry into blood vessels [15] and comprehensive transcriptomic analysis of CTC–WBC cluster in BC patients indicated that CTCs associated with neutrophils promoted cell cycle progression, leading to more efficient metastasis formation [16]. Finally, neutrophil extracellular traps (NETs) were shown to help CTCs to spread and adhere to distant sites [17]. Interestingly, in mice, NETs that were formed during lung inflammation could induce awakening of cancer cells [18].

Whereas no data are available for the interaction between pro-inflammatory blood cells and DTCs in BC, two studies evaluated the association with CTCs. In metastatic BC, the presence of five or more CTCs in 7.5 mL blood significantly correlated positive with percentages of higher neutrophils and monocytes, however, in multivariate analysis, only monocytes were associated with ≥5 CTC. In multivariable analysis for predictors of OS, CTCs, the number of metastatic sites, tumor subtypes and MLR remained significant [19]. In early staged diagnosed BC, patients harboring CTCs in EMT and an NLR above a threshold of three had an 8.6 times increased risk of recurrence compared with CTC-EMT-negative patients and a NLR < 3 [20]. 

In order to analyze the relationship between immune cell ratios and tumor cells more comprehensively, we here evaluated the correlation of NLR, PLR, MLR and the presence of epithelial CTCs (eCTCs), EMT-CTCs as well as DTCs in 171 early staged diagnosed, non-metastatic BC patients before the start of adjuvant treatment to better identify patients at risk and probably adjust therapeutic options.

## 2. Results

### 2.1. Clinical Characteristics of Patients

The clinical characteristics of all chemo-naive, early staged diagnosed, non-metastatic BC patients (*n* = 171) at the time of primary diagnosis are shown in Table 1. The median age of the patients was 61 years (range 31–83 years). The majority of the patients were post-menopausal (71.9%), had T1 (59.6%) and T2 (37.4%) tumors, 59.1% were node-negative (38.6% = N1) and most of the patients presented with a poor or moderately (GII and III) differentiated tumor (93.6%). Expression of the estrogen (ER) and progesterone receptor (PR) was observed in 78.4% and 69.6% of the tumors and human epidermal growth factor receptor (HER)2 was overexpressed in 15.8% of cases, respectively. When patients were stratified according to their histological subtypes, 69% were ER- and or PR-positive and HER2-negative, 15.2% were triple-negative, 11.1% triple-positive and 4.7% showed HER2 overexpression (ER and PR-negative), respectively. 58/170 (34.1%) of the patients were DTC-positive and 44/155 (28%) of the patients were CTC-positive, respectively. 

### 2.2. Establishment of Cut-Off Values

**Figure 1:**
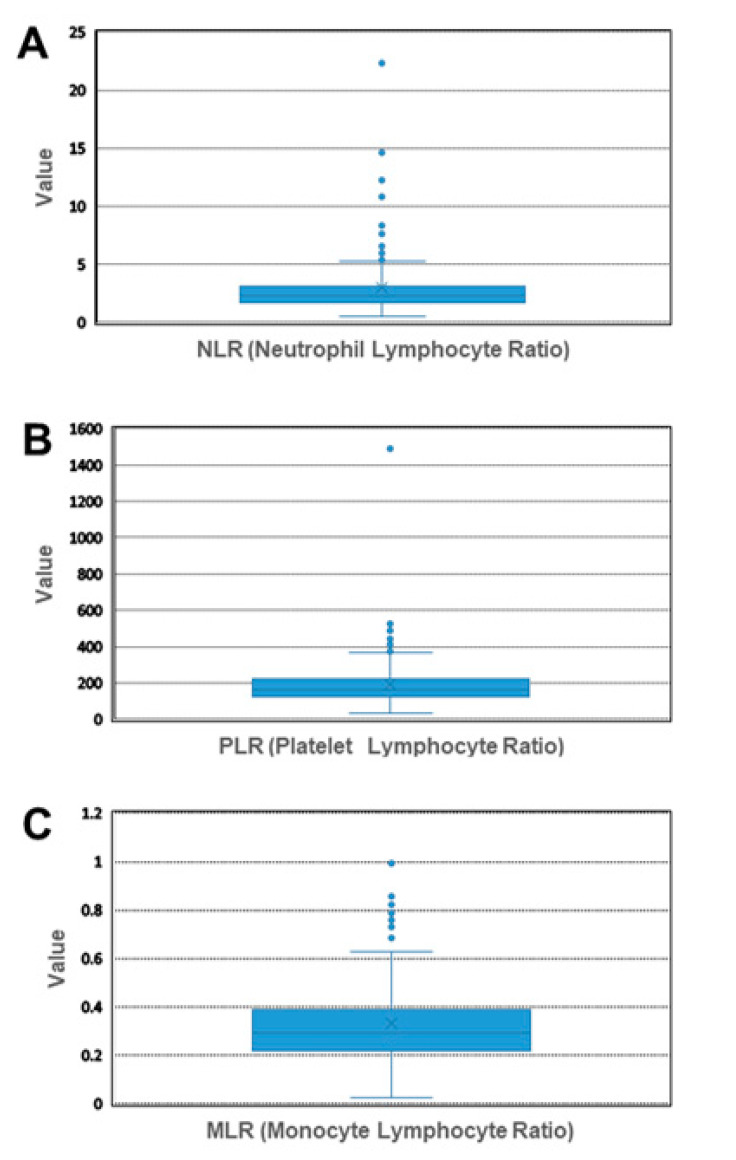
Box and Whisker plot of blood values. Cut-off levels for NLR (**A**), PLR (**B**) and MLR (**C**) of all patients, as determined by the 75% percentile. MLR: monocyte-to-lymphocyte ratio; NLR: neutrophil-to-lymphocyte ratio; PLR: platelet-to-lymphocyte ratio.

NLR, PLR and MLR were calculated from peripheral blood cell counts (Table 2) and their cut-off levels were determined by the 75% percentile resulting in the values 3.13 for NLR, 222.3 for PLR and 0.39 for MLR, respectively (Figure 1).

### 2.3. Correlation of NLR, PLR and MLR with Clinical Characteristics

When patients characteristics as well as DTCs and CTCs were correlated with NLR, MLR and PLR, only the lymph node status was significantly related to PLR (*p* = 0.029) and MLR (*p* = 0.041) while CTCs significantly related to NLR (*p* = 0.025) (Table 3). In more detail, patients with a high NLR (≥3.13) level had significantly more often a positive CTC-status. Patients with a high PLR (≥222.3) or high MLR (≥0.39) level, had significantly more often a pN2-status.

### 2.4. Mean Differences of CTC Subtypes, DTCs and Blood Values

**Figure 2:**
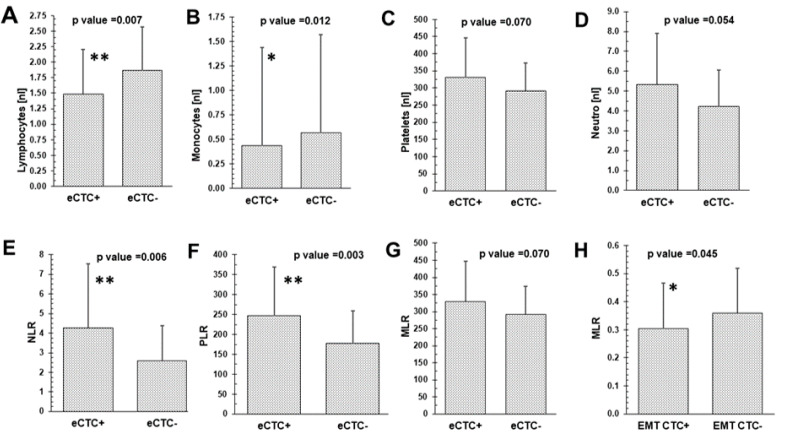
Blood values grouped according to the presence/absence of different CTC subtypes. Differences of median with Mann–Whitney U-test of eCTCs with lymphocytes (**A**), monocytes (**B**), platelets (**C**), neutrophils (**D**), NLR (**E**), PLR (**F**) and MLR (**G**). Differences of median with Mann–Whitney U-test of EMT-CTCs with MLR (**H**). CTCs: epithelial circulating tumor cells; EMT-CTCs: epithelial–mesenchymal transition-circulating tumor cells. MLR: monocyte-to-lymphocyte ratio; NLR: neutrophil-to-lymphocyte ratio; PLR: platelet-to-lymphocyte ratio. * *p* > 0.05; ** *p* < 0.01.

Epithelial CTCs (eCTCs) were detected in 17% (27/155), EMT-CTCs in 42% (33/79) and at least one of the two CTC subtypes in 28% (44/155) of the patients, respectively. As shown in Figure 2, the presence of eCTCs significantly related to reduced lymphocyte (*p* = 0.007) and monocyte counts (*p* = 0.012) as well as an elevated NLR (*p* = 0.006) and PLR (*p* = 0.001). In contrast, the presence of EMT-CTCs only correlated with a significantly reduced MLR (*p* = 0.045). No significant correlations were found for blood cell counts, NLR, PLR and MLR with DTCs.

### 2.5. Prognostic Role of Blood Cell Counts, Ratios, CTCs and DTCs

**Figure 3:**
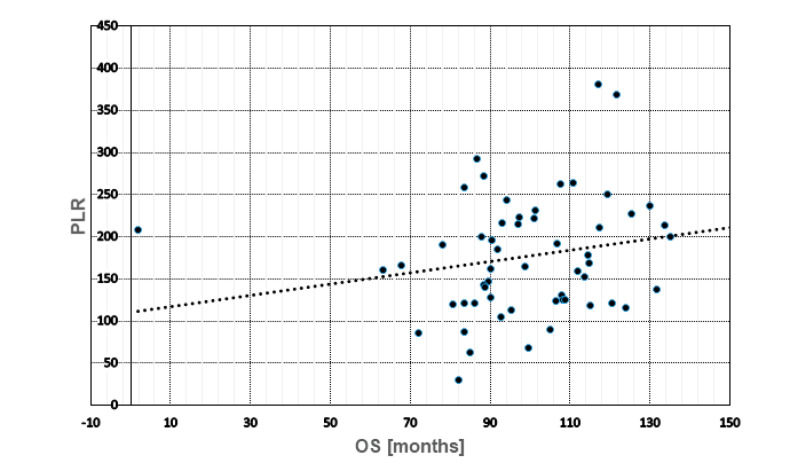
Scatterplot with regression line between blood cells and DTCs. No significant correlations were found for NLR, PLR and MLR with DTCs; however, DTC-positive patients, harboring a lower PLR, had significantly shorter OS (*p* = 0.043).

**Figure 4:**
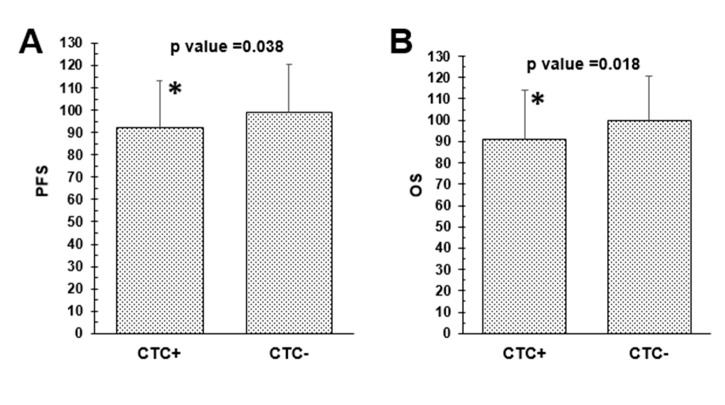
Differences of median with Mann–Whitney U-test of CTCs with outcome. The presence of CTCs was significantly different for shorter progression-free survival (*p* = 0.038) and overall survival (*p* = 0.018). * *p* < 0.05.

Using Spearman-Rho Test (Table 4), no significant correlations with regard to PFS and OS were found for neutrophils, lymphocytes, platelets, NLR, PLR and MLR, respectively. Nevertheless, high monocyte counts significantly related to a reduced OS (*p* = 0.034). Whereas the presence of DTCs did not correlate with PFS (*p* = 0.254) or OS (*p* = 0.185), DTC-positive patients, harboring a lower PLR, had a significant shorter OS (*p* = 0.043) (Figure 3). The presence of CTCs was significantly correlated with a shorter PFS (*p* = 0.038) and OS (*p* = 0.018) (Figure 4), however, neither eCTCs nor EMT-CTCs alone were of prognostic significance. In patients still alive, a reduced PFS/OS significantly correlated with enhanced lymphocyte (*p* = 0.025/0.011) and monocyte counts (*p* = 0.039/*p* = 0.037) as well as a low PLR (*p* = 0.032/0.023) whereas an enhanced MLR showed a high correlation with a shorter PFS (*p* = 0.007) and OS (*p* = 0.021) in deceased patients (data not shown).

### 2.6. Prognostic Value of CTCs, DTCs in Combination with NLR, PLR and MLR

For all specific combinations tested, e.g., CTC-positive & NLR ≥3.13, CTC-positive & NLR <3.13, CTC-negative & NLR ≥3.13, CTC-negative & NLR <3.13, no significant correlations with regard to PFS (*p* = 0.570) and OS (*p* = 0.473) were documented. PFS and OS values for the relationship between CTCs and PLR were *p* = 0.918/*p* = 0.909, CTCs and MLR *p* = 0.296/*p* = 0.561, DTC and NLR *p* = 0.289/0.524 DTCs and PLR *p* = 0.377/0.369 and DTCs and MLR *p* = 0.288/*p* = 0.287, respectively (Supplementary Figure S1).

### 2.7. Univariate and Multivariate Analysis

As apparent from Table 5, in univariate analysis, good prognostic factors with regard to PFS and OS were age >60 years (PFS: *p* = 0.046; OS: *p* = 0.027) and no lymph node involvement (PFS: *p* = 0.047; OS: *p* = 0.020). For multivariate analysis, patients who presented with a N0 status, OS was close to significance (*p* = 0.050). For all other variables including NLR, PLR, MLR, CTCs and DTCs, no significant relationships were found (Table 5).

## 3. Discussion

Although early staged diagnosed BC is considered potentially curable and therapeutic options have substantially progressed over the last years, it is estimated that 20–30% of all BC cases will become metastatic, even after 10–20 years. In addition, BC prognosis depends on a variety of patient and tumor factors, however, markedly different clinical outcomes are seen among patients with similar prognostic factors [1]. Thus, research in early staged diagnosed BC currently aims to discover new biomarkers to better estimate patients‘ clinical outcome, identify patients who might have a higher risk for relapse and to get insights to adjust treatment.

In this regard, the presence and persistence of CTCs as well as DTCs were shown to be of prognostic significance [4,21] which is also confirmed in our study for CTCs with regard to PFS and OS while DTCs showed no prognostic significance. The latter finding goes in line with our previously published studies which explain these results with the intake of bisphosphonates in case of DTC-positivity which was shown to markedly improve the outcome of our patients [22,23,24,25].

However, while travelling from the primary site into secondary organs, tumor cells leave their original surrounding, loose contact to epithelial cells and have to adapt to cells in the blood stream where they are directly exposed to the immune system which should, in principle, try to eliminate them [26]. Although inflammatory cells and mediators in the tumor microenvironment are thought to play an important role in cancer progression, the interplay with CTCs and DTCs in early staged diagnosed BC has rarely been addressed [27].

The prognostic and predictive value of pro-inflammatory markers including ratios to lymphocytes has often been studied in BC patients receiving neo-adjuvant chemotherapy with mainly neutrophils as well as NLR in their focus [12,28,29]. Pre-treatment values of NLR and MLR in these patients were shown to be suitable biomarker for predicting treatment efficacy, pathological complete remission and survival [30,31,32,33]. Up to now, only a very few studies have focused on early staged diagnosed BC. In our study, we could not demonstrate any significances for neutrophils, lymphocytes, platelets, NLR, PLR and MLR with regard to PFS and OS, respectively. This is in contrast to the other published articles, however, it is difficult to compare these studies because different cut-off values, in general lower values, for all ratios were used [34]. In this context, a cut-off above 1.8 for NLR was shown to relate to a postoperative recurrence [35], above 1.97 to a significant worse prognosis [36] and above 2.65 to a shorter OS, repectively [37]. Referring to meta analyses including early staged diagnosed and locally advanced BC patients, the most frequently used cut-off for NLR was 3.0 (as median) which is similar to the value we used (3.13 as 75% percentile) [34]. Applying exactly the cut-off of 3.0, only NLR among several other immune–based scores tested, was significantly related to a reduced PFS in a cohort of 284 early staged diagnosed BC patients [20].

The prognostic potential of PLR and MLR was identified less frequently in early staged diagnosed BC. Published PLR scores of >185 and >190.9 (thus, below our applied score of 222.3 with no significant results obtained for PFS and OS), were related to a shorter OS in univariate analyses [37,38]. In contrast, high monocyte counts in our patient cohort significantly correlated with a reduced OS which goes in line with a large cohort study, which demonstrated that elevated pre-operative circulating absolute monocyte counts were related to a shorter OS [39]. For MLR, using a cut-off of 0.39, no significant relationships were found for PFS and OS which was confirmed in a comprehensive study in early staged diagnosed BC applying a cut-off of 0.34 [20].

For the interplay of immune cells with tumor cells, our retrospective analysis confirms that pro-inflammatory markers in blood, namely NLR, PLR and MLR, are closely related to the presence of different CTC subtypes. While eCTCs were closely related to reduced lymphocyte and monocyte counts and an elevated NLR and PLR, the presence of EMT-CTCs significantly correlated with a reduced MLR. In addition, DTC-positive patients with a documented lower PLR had a significant shorter OS. With regard to CTCs, only a few studies have addressed this topic in solid tumors including BC. Zheng et al., correlated pre-operative inflammation-based indexes, including a systemic immune-inflammation index (SII), NLR, PLR as well as a prognostic nutrition index (PNI), with CTC counts in 60 patients with gastric cancer. Selecting epithelial tumor cells by size using ISET (Isolation by SizE of Tumor Cells) with subsequent staining of the cells with ostom Y and methylene blue for Wright staining, they demonstrated that NLR and PLR were clinically meaningful to predict eCTC counts and eCTC detection ratios and the correlation suggested that improved scores of NLR and PLR were consistent with increased eCTC counts [40]. These data are, somehow, comparable with our finding and emphasize that the analysis of different CTC subtypes might help to improve the prognostication of patients. In a retrospective analysis of 516 metastatic BC patients, eCTC counts obtained with the CellSearch System significantly correlated with the number of monocytes and neutrophils and in a multivariate analysis, in combination with a MLR ≥0.34, eCTCs were predictors of a decreased OS [19]. Comparable with our study, Miklikova et al., evaluated NLR, MLR, PLR and SIL (systemic immune-inflammation index), referred to as complete blood count (CBC)-derived inflammation-based scores, in 284 early BC patients and correlated these findings with the EMT-CTC subtype. Using CD45 depletion (RossetteSep^TM^), followed by reverse transcription of enriched mRNA to detect the EMT-related transcription factors *TWIST1, SNAIL1*, *SLUG*, and *ZEB1* gene transcripts, a strong relationship between adverse outcome and elevated NLR and MLR in EMT-CTC-positive patients was observed. They concluded that their findings support the hypothesis that immune cells in the bloodstream can expand the metastatic potential of CTCs [20]. Although different methods were used to detect CTC subtypes in these studies, it seems as if NLR, PLR and MLR are closely related to the presence of CTC subtypes, even in early BC patients where CTCs, in general, are rare [21].

The interplay between CTCs/DTCs and the tumor microenvironment is complex and poorly understood. A variety of important infiltrating and circulating immune cells hinder or favor the dissemination of CTCs, including natural killer cells, T-cells, neutrophils, monocytes and macrophages as well as platelets [27]. In this regard, a variety of mechanisms have been hypothesized through which CTCs escape and survive. In this context, some of the CTCs are known to express CD47, a ‘don′t eat me signal’ and PD-L1 has been detected on BC CTCs which prevents T-cell mediated destruction [41,42,43]. Furthermore, CTCs are frequently present inside circulating tumor micro-emboli or white blood cell clusters (WBCs) which protect them from being recognized [44]. Among all immune cells discussed to support CTCs, neutrophils seem to play a major role. Besides NETs that help CTCs to spread and adhere to distant sites [17], CTCs were shown to co-localize with neutrophils in the pre-metastatic vascular network, suggesting that neutrophils can retain cancer cells and facilitate their extravasation. A recently published study showed that WBCs–CTC clusters of BC patients, although quite rare, were related to neutrophils [16]. Interestingly, the transcriptomic profiles of CTCs associated with neutrophils were different from those of single CTCs with regard to cell cycle progression, thus, probably leading to metastasis. These data also support our study which documents a significant relationship between NLR and eCTCs. In addition, CTC clusters detected in advanced BC exhibited mesenchymal features and showed attached CD61-positive platelets that are known to induce EMT-like features in CTCs [45]. We did not find significant relationships between platelets and CTC subtypes, however, an enhanced PLR was closely related to the presence of eCTCs but not to EMT-CTCs which in turn were correlated with a reduced MLR. We can only speculate about the cause of the latter finding. In general, circulating monocytes can extravasate and differentiate into macrophages with pro-tumor and pro-metastatic functions. In this context, Zhang et al., showed that a subgroup of macrophages was able to phagocyte CTCs, incorporate their content and thus, express malignant features [46]. Furthermore, tumor associated macrophages were shown to facilitate cancer cell migration by activating EMT [47] which might partly explain our findings.

To the best of our knowledge, no data have been published with regard to the relationship between pro-inflammatory markers in blood and their relationship to DTCs that settle down in the BM. Our current study did not identify significant correlations between blood cell counts, NLR, PLR, MLR and DTCs, however, DTC-positive patients with a documented lower PLR had a significant shorter OS. For homing of DTCs in secondary organs, other immune cells or factors than those we evaluated, might be relevant. In this context, the chemokine receptor CXCR4 has been favored to help tumor cells to settle down in the BM [48] and we recently demonstrated that CXCR4/JUNB/CK-expressing DTCs were frequently detected in the BM of early staged diagnosed BC patients and seem to identify a subgroup of patients at higher risk for relapse [10].

## 4. Patients and Methods

### 4.1. Study Population

This retrospective study was conducted in the Department of Gynecology and Obstetrics at the University Hospital of Essen and included 171 early staged diagnosed, non-metastatic BC patients who presented with first diagnosis of BC between July 2006 and December 2012. The eligibility criteria were as follows: histologically proven BC, no severe uncontrolled co-morbidities or medical conditions, no further malignancies at present or in history, completion of adjuvant treatment according to guidelines including adjuvant chemotherapy (anthracyclines, 5-fluorouracil, taxanes, cyclophosphamide), anti-hormonal therapy in case of hormone responsive tumors (tamoxifen or an aromatase inhibitor), trastuzumab in case of HER2-positivity and radiotherapy. All DTC-positive patients were recommended an additional therapy with oral clodronate (2 × 520 mg per day for at least two years). Patients treated with neo-adjuvant chemotherapy were excluded.

All specimens were obtained after written informed consent from all subjects prior to inclusion in the study and collected using protocols approved by the clinical Ethic committee of the University Hospital Essen (05/2856). All methods were carried out in accordance with the approved guidelines.

From a total number of 623 early staged diagnosed BC patients, patients’ selection for this study was based on the availability of differential blood cell counts at primary diagnosis, before any treatment. 171/623 BC patients fulfilled these criteria and for these patients, DTC as well as CTC analysis was performed at the same time point, before the start of any therapy.

After a median follow-up time of 96.3 months (range: 1.8 to 152 months and 95% CI was 93.0 to 99.5 month). The OS rate was 91.2% (156/171 patients) and relapses occurred in 5.84% (10/171 patients) of cases.

### 4.2. Selection and Detection of CTCs

CTCs were isolated from 4 × 5 mL blood drawn before the application of therapeutic substances and before surgery with an S-Monovette (Sarstedt AG & Co., Nümbrecht, Germany) and stored at 4 °C until further examination. The samples were processed immediately or latest four hours after blood withdrawal. Establishment and validation of this assay has been described in detail elsewhere [24]. Briefly, CTCs were selected by positive immune-magnetic selection targeting EpCAM and MUC1 using the AdnaTest *BreastCancerSelect* (QIAGEN, Hilden, Germany), followed by mRNA isolation from lysed, enriched cells and subsequent reverse transcription resulted in cDNA, which was the template for tumor cell detection and characterization by multiplex PCR. eCTCs were determined applying the AdnaTest *BreastCancerDetect* (QIAGEN, Hilden, Germany) for gene expression analysis of *EpCAM* (*GA733-2*), *MUC-1* and *HER2 (ERBB2)*. The test is considered positive if an amplicon of at least one tumor-associated transcript was detected over the threshold defined in the handbook. EMT-CTCs were detected using the AdnaTest *EMT Detect* (QIAGEN, Hilden, Germany) for the expression of *PI3K*, *AKT* and *TWIST*. The test is considered positive if an amplicon of at least one tumor-associated transcript was detected over the threshold defined in the handbook. We defined a patient CTC positive if one of the two tests were positive. Visualization of the PCR fragments was carried out with a 2100 Bio-Analyzer using the DNA 1000 LabChips (Agilent Technologies) and the Expert Software Package (version B.02.03.SI307) both Santa Clara, CA, USA.

### 4.3. Selection and Detection of DTCs

Between 10 and 20 mL BM were aspirated from the anterior iliac crests of all patients at the beginning of surgery of the primary tumor, before start of any therapy and processed within 24 h. BM tumor cell isolation and detection have been described elsewhere [22,23,24,25]. Briefly, BM cells were isolated from heparinized BM (5000 U/mL BM) by Ficoll-Hypaque density gradient centrifugation (density 1.077 g/mol; Pharmacia, Freiburg, Germany) at 400× *g* for 30 min. Slides were analyzed for DTCs by immunocytochemistry using the pan-cytokeratin antibody A45-B/B3. Microscopic evaluation of the slides was carried out using the ARIOL system (Applied Imaging). A patient was defined as DTC-positive in case one cytokeratin-positive cell was detected.

### 4.4. Statistical Analysis

Statistical analysis included descriptive reporting, U-test for group differences, Rho correlations for dependencies and Fisher’s chi-square test for distributional differences. Rho correlations were used to assess the relationship between NLR, MLR, PLR and tumor cells as well as PFS and OS. We utilized cox regression analysis to generate a hazard ratio with confidence intervals for OS and PFS and a variety of markers and clinical parameters.

## 5. Limitations of the Study and Conclusions

The relationship between tumor cells and their environment is very complex, however, might be promising to develop new treatment strategies in the future. The strength of our study is reflected by significant relations between CTC subtypes and blood cell ratios which encourages to get deeper insights into the origin of circulating neutrophils, platelets, monocytes as well as T- and B-cells in the future. However, the study also has some limitations. Non-consecutive patients, undergoing adjuvant treatment were enrolled until about 10 years ago, and the patient population largely consisted of hormone receptor-positive, HER2-negative patients. Consequently, the results are applicable almost exclusively to this BC subgroup. Upcoming studies in this direction should include other BC subgroups, mostly receiving neoadjuvant chemotherapy nowadays as well as more sampling time points to draw more precise conclusions.

Among all markers evaluated up to now, neutrophils and NLR seem to be the most promising markers and could serve as a predictor of patients’ survival to help with treatment decisions. Although therapeutic options are rarely available right now, interesting experimental studies demonstrated that blocking the interaction of tumor cells and platelets [49,50] or antibodies against NET-remodeled laminin, that prevent awakening of dormant cells, which might be a promising approach in the future [18].

## Figures and Tables

**Table 1:** Patients characteristics at the time of first diagnosis.

	Number of Patients	%
**Age (years)**		
Median	61	
Range	31–83	
**Menopausal Status**		
Premenopausal	32	18.7
Perimenopausal	16	9.4
Postmenopausal	123	71.9
**Histology**		
Ductal	134	78.4
Lobular	17	9.9
Others	20	11.7
**Tumor Size**		
pT1	102	59.6
pT2	64	37.4
>pT2	5	2.9
**Nodal Status**		
pN0	101	59.1
pN1	66	38.6
pN2 and pN3	4	2.3
**Grading**		
I	11	6.4
II	100	58.5
III	60	35.1
**ER Status**		
Negative	37	21.6
Positive	134	78.4
**PR Status**		
Negative	52	30.4
Positive	119	69.6
**HER2 Status**		
Negative	144	84.2
Positive	27	15.8
**Immunohistochemical Subtype**		
(ER−, PR−, HER2−)	26	15.2
(ER−, PR−, HER2+)	8	4.7
(ER+ and/or PR+, HER2−)	118	69
(ER+ and/or PR+, HER2+)	19	11.1
**DTC**		
Negative	112	65.9
Positive	58	34.1
n.d.	1	
**CTC**		
Negative	111	72
Positive	44	28
n.d.	16	
**NLR**		
>3.13	42	25
<3.13	127	75
n.d.	2	
**PLR**		
>222.3	42	25
<222.3	129	75
**MLR**		
>0.39	47	28
<0.39	122	72
n.d.	2	
**Recurrent**	**10**	**6**
**Deceased**	**15**	**9**

**Table 2:** Cut-off values for peripheral blood cell counts and their ratios. Cut-off levels for blood values of all patients were determined by the 75% percentile (gray column). MLR: monocyte-to-lymphocyte ratio; NLR: neutrophil-to-lymphocyte ratio; PLR: platelet-to-lymphocyte ratio.

	*n*	Mean	Median	Perc 25	Perc 75	SD	Min	Max
NLR	169	2.97	2.24	1.68	3.13	2.6	0.45	22.37
PLR	170	193.6	161	124.5	222.3	135	30.2	1492.7
MLR	169	0.33	0.29	0.22	0.39	0.17	0.03	0.99
Neutrophils (nL)	169	4.45	4.03	3.13	5.31	2	0.25	11.8
Lymphocytes (nL)	170	1.81	1.7	1.34	2.2	0.71	0.41	4.3
Platelets (nL)	171	296.6	283	237	336	87.3	52	612
Monocytes (nL)	169	0.55	0.53	0.4	0.67	0.23	0.02	1.36
Age (y)	171	58.6	61	50	68	11.3	31	83

**Table 3:** Correlation of NLR, PLR and MLR with clinical characteristics. CTCs: circulating tumor cells; DTCs: disseminated tumor cells; ER: estrogen receptor; HER2: human epidermal growth factor receptor 2; MLR: monocyte-to-lymphocyte ratio; NLR: neutrophil-to-lymphocyte ratio; PLR: platelet-to-lymphocyte ratio; PR: progesterone receptor; pN: lymph node involvement; pT: tumor size. ^$^ The test for significant differences in distribution was performed using the chi-square test.

		NLR (Cut-Off)				Sig ^$^	PLR (Cut-Off)				Sig ^$^	MLR (Cut-Off)				Sig ^$^
		≥3.13		<3.13			≥222.3		<222.3			≥0.39		<0.39		
		*n*	%	*n*	%	*p*-Value	*n*	%	*n*	%	*p*-Value	*n*	%	*n*	%	*p*-Value
**Age Grouped (60 y)**	<60	25	58.1	66	52.4	0.513	25	58.1	67	52.8	0.54	26	61.9	65	51.2	0.227
	≥60	18	41.9	60	47.6		18	41.9	60	47.2		16	38.1	62	48.8	
	Total	43	100	126	100		43	100	127	100		42	100	127	100	
**Menopausal** **Status**	Premenopausal	9	20.9	22	17.5	0.399	8	18.6	23	18.1	0.997	7	16.7	24	18.9	0.426
	Perimenopausal	6	14	10	7.9		4	9.3	12	9.4		2	4.8	14	11	
	Postmenopausal	28	65.1	94	74.6		31	72.1	92	72.4		33	78.6	89	70.1	
	Total	43	100	126	100		43	100	127	100		42	100	127	100	
**Histology**	Ductal	30	69.8	102	83.6	0.128	31	73.8	102	82.3	0.478	30	75	102	81.6	0.657
	Lobular	6	14	11	9		6	14.3	11	8.9		5	12.5	12	9.6	
	Other	7	16.3	9	7.4		5	11.9	11	8.9		5	12.5	11	8.8	
	Total	43	100	122	100		42	100	124	100		40	100	125	100	
**pT**	pT1	26	60.5	74	58.7	0.951	27	62.8	74	58.3	0.86	21	50	79	62.2	0.107
	pT2	16	37.2	48	38.1		15	34.9	49	38.6		18	42.9	46	36.2	
	pT3 pT4	1	2.3	4	3.2		1	2.3	4	3.1		3	7.1	2	1.6	
	Total	43	100	126	100		43	100	127	100		42	100	127	100	
**pN**	pN0	22	51.2	78	61.9	0.45	18	41.9	82	64.6	**0.029**	18	42.9	82	64.6	**0.041**
	pN1	20	46.5	45	35.7		24	55.8	42	33.1		23	54.8	42	33.1	
	pN2 pN3	1	2.3	3	2.4		1	2.3	3	2.4		1	2.4	3	2.4	
	Total	43	100	126	100		43	100	127	100		42	100	127	100	
**Grading**	G1	1	2.3	9	7.1	0.254	2	4.7	8	6.3	0.568	1	2.4	9	7.1	0.338
	G2	23	53.5	76	60.3		23	53.5	77	60.6		23	54.8	76	59.8	
	G3	19	44.2	41	32.5		18	41.9	42	33.1		18	42.9	42	33.1	
	Total	43	100	126	100		43	100	127	100		42	100	127	100	
**ER Status**	No	13	30.2	23	18.3	0.098	10	23.3	27	21.3	0.784	9	21.4	27	21.3	0.982
	Yes	30	69.8	103	81.7		33	76.7	100	78.7		33	78.6	100	78.7	
	Total	43	100	126	100		43	100	127	100		42	100	127	100	
**PR Status**	No	15	34.9	36	28.6	0.436	14	32.6	38	29.9	0.746	12	28.6	39	30.7	0.794
	Yes	28	65.1	90	71.4		29	67.4	89	70.1		30	71.4	88	69.3	
	Total	43	100	126	100		43	100	127	100		42	100	127	100	
**HER2 Status**	No	35	81.4	107	84.9	0.586	33	76.7	110	86.6	0.126	33	78.6	109	85.8	0.266
	Yes	8	18.6	19	15.1		10	23.3	17	13.4		9	21.4	18	14.2	
	Total	43	100	126	100		43	100	127	100		42	100	127	100	
**ER– PR– HER2–**	No	34	79.1	110	87.3	0.189	35	81.4	109	85.8	0.489	36	85.7	108	85	0.915
	Yes	9	20.9	16	12.7		8	18.6	18	14.2		6	14.3	19	15	
	Total	43	100	126	100		43	100	127	100		42	100	127	100	
**ER– PR– HER2+**	No	40	93	121	96	0.422	41	95.3	121	95.3	0.984	39	92.9	122	96.1	0.396
	Yes	3	7	5	4		2	4.7	6	4.7		3	7.1	5	3.9	
	Total	43	100	126	100		43	100	127	100		42	100	127	100	
**ER + AND (PR + OR Her2–)**	No	13	30.2	29	23	0.344	11	25.6	32	25.2	0.96	10	23.8	32	25.2	0.857
	Yes	30	69.8	97	77		32	74.4	95	74.8		32	76.2	95	74.8	
	Total	43	100	126	100		43	100	127	100		42	100	127	100	
**ER + AND (PR + OR Her2+)**	No	16	37.2	32	25.4	0.138	13	30.2	36	28.3	0.813	11	26.2	37	29.1	0.714
	Yes	27	62.8	94	74.6		30	69.8	91	71.7		31	73.8	90	70.9	
	Total	43	100	126	100		43	100	127	100		42	100	127	100	
**DTC Status**	Neg	29	67.4	82	65.6	0.826	28	65.1	84	66.7	0.853	24	57.1	87	69	0.158
	Pos	14	32.6	43	34.4		15	34.9	42	33.3		18	42.9	39	31	
	Total	43	100	125	100		43	100	126	100		42	100	126	100	
**CTC Status**	Neg	23	57.5	86	76.1	**0.025**	26	61.9	84	75	0.109	25	67.6	84	72.4	0.571
	Pos	17	42.5	27	23.9		16	38.1	28	25		12	32.4	32	27.6	
	Total	40	100	113	100		42	100	112	100		37	100	116	100	

**Table 4:** Correlation between blood cells, ratios and outcome. MLR: monocyte-to-lymphocyte ratio; NLR: neutrophil-to-lymphocyte ratio; OS: overall survival; PFS: progression-free survival; PLR: platelet-to-lymphocyte ratio. Spearman’s rho test: values < 0.05 are significant.

		NLR	PLR	MLR	Neutrophils (nL)	Lymphocytes (nL)	Platelets (nL)	Monocytes (nL)
PFS	r	0.073	0.143	−0.067	−0.096	−0.130	0.101	0.144
	*p*	0.350	0.066	0.392	0.221	0.094	0.193	0.065
	*n*	165	166	165	165	166	167	165
OS	r	0.053	0.150	−0.085	−0.085	−0.146	0.083	−0.163
	*p*	0.497	0.050	0.271	0.164	0.058	0.283	**0.034**
	*n*	169	170	169	169	170	171	169

**Table 5:** Cox proportional hazards regression (HR) model for univariate and multivariate analyses for the ratios, clinical characteristics and outcome. CTCs: circulating tumor cells; DTCs: disseminated tumor cells; MLR: monocyte-to-lymphocyte ratio; NLR: neutrophil-to-lymphocyte ratio; OS: overall survival; PFS: progression-free survival; PLR: platelet-to-lymphocyte ratio.

	OS				PFS			
	Univariate		Multivariate		Univariate		Multivariate	
	HR (95% CI)	*p*-Value	HR (95% CI)	*p*-Value	HR (95% CI)	*p*-Value	HR (95% CI)	*p*-Value
**Age (years)**								
< vs. > 60	4.195 (1.182–14.892)	**0.027**	2.801 (0.552–14.203)	0.214	4.784 (1.032–22.181)	**0.046**	2.355 (0.375–14.782)	0.361
**Tumor size**								
T1 vs. >T1	2.182 (0.775–6.139)	0.139	2.159 (0.672–6.932)	0.196	2.533 (0.740–8.674)	0.137	2.916 (0.669–12.705)	0.154
**Lymph node** **involvement**								
N0 vs. N+	3.889 (1.238–12.216)	**0.020**	3.423 (0.999–11.730)	**0.050**	3.836 (1.017–14.465)	**0.047**	3.216 (0.752–13.746)	0.115
**Menopausal Status**								
Pre- and peri vs. postmenopausal	2.785 (0.627–12.359)	0.178	1.181 (0.169–8.235)	0.866	4.266 (0.545–33.369)	0.167	2.165 (0.185–25.378)	0.538
**NLR**								
< vs. ≥3.13	1.909 (0.678–5.373)	0.221	2.073 (0.546–7.870)	0.284	2.336 (0.711–7.667)	0.162	2.696 (0.555–13.089)	0.219
**PLR**								
< vs. ≥222.3	0.671 (0.189–2.382)	0.537	0.303 (0.069–1.326)	0.113	0.608 (0.131–2.820)	0.525	0.281 (0.049–1.618)	0.155
**MLR**								
< vs. ≥0.39	1.639 (0.559–4.805)	0.368	1.500 (0.419–5.367)	0.533	1.964 (0.572–6.745)	0.284	1.575 (0.354–7.013)	0.551
**CTCs**								
Pos. vs. Neg.	1.151 (0.360–3.677)	0.813	0.981 (0.294–3.269)	0.975	1.194 (0.308–4.628)	0.797	0.904 (0.218–3.751)	0.890
**DTCs**								
Pos. Vs. neg.	1.172 (0.416–3.305)	0.764	0.825 (0.251–2.716)	0.752	1.467 (0.446–4.828)	0.528	0.959 (0.240–3.833)	0.953

## Data Availability

Raw data can be provided upon request to the corresponding author.

## Supplementary Materials

The following supporting information can be downloaded at: https://www.mdpi.com/article/10.3390/cancers14143299/s1, Figure S1: Kaplan-Meier PFS/OS estimates for all combinations of CTCs/DTCs and ratios tested.
