# Supplementary material for: In Early Breast Cancer, the Ratios of Neutrophils, Platelets and Monocytes to Lymphocytes Significantly Correlate with the Presence of Subsets of Circulating Tumor Cells but Not with Disseminated Tumor Cells
Source: Cancers (Basel). 2022 Jul 6;14(14):3299. doi: 10.3390/cancers14143299 (PMC9320225; doi:10.3390/cancers14143299)
Supplement: Supplementary file 1 [file cancers-14-03299-s001.zip › cancers-1779396_supplementary.pdf]

cancers

MDPI

Article

In early breast cancer, the ratios of neutrophils, platelets and monocytes to lymphocytes significantly correlate with the presence of subsets of circulating tumor cells but not with disseminated tumor cells.

Sabine Kasimir-Bauer[1,*], Ebru Karaaslan[1], Olaf Hars[2], Oliver Hoffmann[1], Rainer Kimmig[1]

K

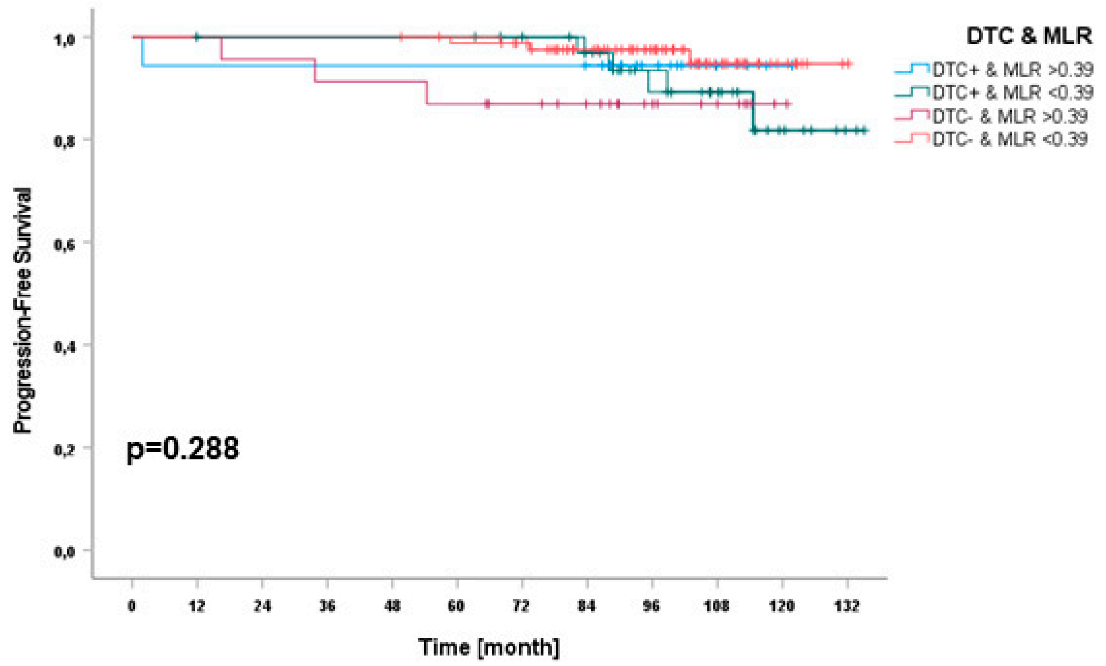

L

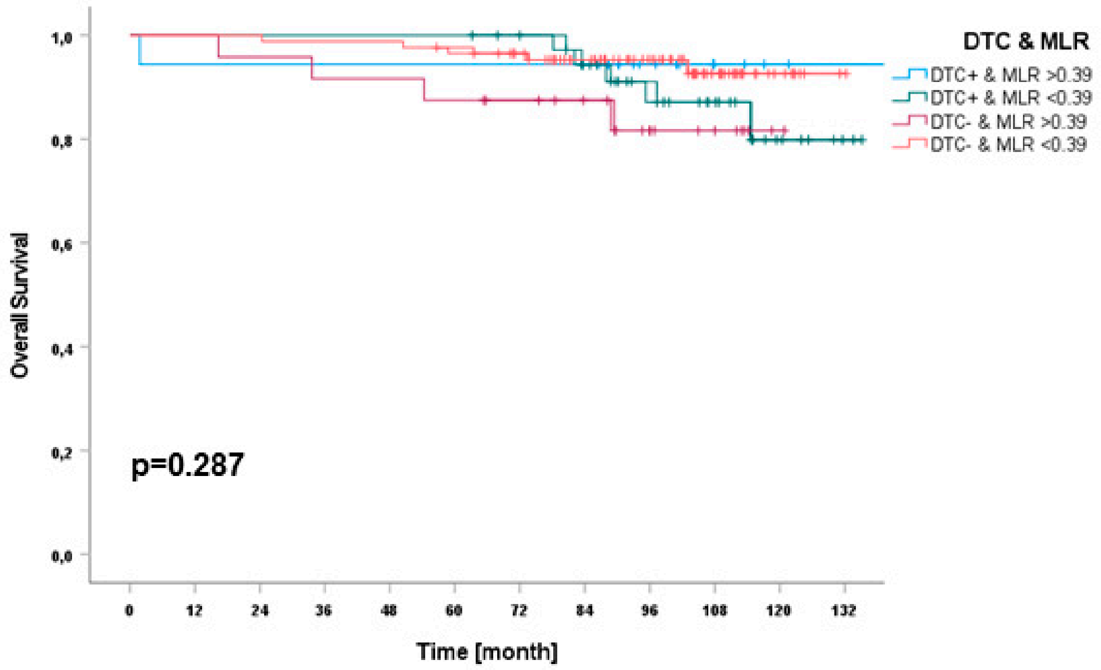

Figure S1. Kaplan-Meier PFS/OS estimates for all combinations of CTCs/DTCs and ratios tested.
